# Changes in Current Transport and Regulation of the Microstructure of Graphene/Polyimide Films under Joule Heating Treatment

**DOI:** 10.3390/ma17112540

**Published:** 2024-05-24

**Authors:** Jianshu Yu, Hui Ding, Bin Chen, Xuejiao Sun, Ying Zhang, Zhongfu Zhou

**Affiliations:** 1 School of Materials Science and Engineering, Shanghai University, Shanghai 200444, China; yu_fae@shu.edu.cn; 2 Inner Mongolia Key Laboratory of Carbon Materials, Baotou 014000, China; dinghui87@gmail.com (H.D.); chenbin_19860113@126.com (B.C.); zhangying90lingyi@163.com (Y.Z.)

**Keywords:** graphene, polyimide, joule heating, composite film, atomic rearrangement

## Abstract

The excellent electrical properties of graphene have received widespread attention. However, the difficulty of electron transfer between layers still restricts the application of graphene composite materials to a large extent. Therefore, in this study, graphene/polyimide films were subjected to a Joule heating treatment to improve the electrical conductivity of the film by ~76.85%. After multiple Joule thermal cycle treatments, the conductivity of the graphene/polyimide film still gradually increased, but the increase in amplitude tended to slow down. Finally, after eight Joule heat treatments, the conductivity of the graphene/polyimide film was improved by ~93.94%. The Joule heating treatment caused the polyimide to undergo atomic rearrangement near the interface bonded to the graphene, forming a new crystalline phase favourable for electron transport with graphene as a template. Accordingly, a model of the bilayer capacitive microstructure of graphene/polyimide was proposed. The experiment suggests that the Joule heating treatment can effectively reduce the distance between graphene electrode plates in the bilayer capacitive micro-nanostructures of graphene/polyimide and greatly increases the number of charge carriers on the electrode plates. The TEM and WAXS characterisation results imply atomic structure changes at the graphene/polyimide bonding interface.

## 1. Introduction

Since the discovery of graphene, its excellent properties have attracted continuous attention, especially the electrical conductivity of monolayer graphene up to 5 × 10^6^ s·m^−1^ [1,2,3]. Nowadays, powdered graphene has been widely used in various applications, including electrodes for energy storage devices [4,5] and conductive fillers [6,7] for polymer nanocomposites. In practice, however, it is often difficult to take advantage of the excellent conductive properties of graphene–polymer composites [8,9]. The main reason for this is that the interfacial contact between graphene sheets impedes electrical transmission, so the conductivity of graphene materials is generally much lower than the intrinsic conductivity of a single graphene sheet [10]. Faced with this problem, some researchers have attempted to introduce similar sp^2^-hybridised covalently bonded carbon atom structures to connect graphene sheets while retaining the original excellent in-plane conductivity of graphene. For example, M. M. Slepchenkov and colleagues used single-walled carbon nanotubes to form single-walled carbon nanotube–graphene junctions of different diameters in graphene nanopores, thereby controlling the conductivity of the material [11]. Another group of researchers built conductive networks using polymer materials as the substrates. For example, J. Y. Suh and his colleagues used the hot pressing method to construct conductive networks with 30 vol% graphene wrapped in polytetrafluoroethylene powder to prepare composites with 7353 s·m^−1^ conductivity [12].

Among the polymers featured in these attempts, polyimide has been used by many researchers as a polymer matrix for graphene composites due to its broad application prospects in the fields of thin-film electrodes [13,14,15], integrated circuit radiators [16,17], high-temperature structural materials [18,19,20] and lithium-ion batteries [21,22]. Moreover, it has been widely used as one of the best pyrolysis precursors for the preparation of carbon films by carbonisation and graphitisation [23,24,25]. R. K. Biswas et al., have thermally converted polyimide to graphene by writing with a pulsed CO_2_ laser on a copper substrate [26]. However, the composites of graphene and polyimide are not ideally dispersed, often requiring dispersants or modification of the graphene to improve its dispersion and binding in the polyimide matrix [27,28]. C. Lim and his colleagues prepared graphene/polyimide films via the electrostatic discharge method and found that the thermal conductivity of the graphene/polyimide films increased exponentially with an increasing graphene content; however, when the graphene content was more than 40 wt%, the graphene/polyimide films could not maintain their shape [29]. On the other hand, Y. Liu et al., synthesised a 2D monolayer polyimide on a water surface and formed a 2D van der Waals heterostructure through its face-to-face assembly with graphene, which achieved ultrafast interlayer charge transfer through interlayer cation-Π interactions between the 2D polyimide and the graphene [30]. Z. Xu and his colleagues prepared a series of flexible electrically heated films using polyimide (PI) as a film-forming substrate and graphene as a conductive filler, with the film with 8 wt% graphene rapidly reaching a temperature of 390 °C under a 24 V power supply [31]. This electrical heating method is called Joule heating [32,33]. P. Zhang et al., prepared polyimide/vapour-grown carbon fibre (VGCF) composites via in situ polymerisation and investigated the frequency–temperature relationship between the Joule thermal modulus and the electron transfer mechanism, concluding that thermal fluctuations in the polyimide chain arrangement are small, despite the collision of the electrons with the atoms of the polyimide chains [34]. Therefore, in the present study, in situ synthesis was used to prepare graphene/polyimide films by the in situ mixing of polyimide precursors with graphene, which ensured the dispersion of the latter. The films were based on a polyimide substrate and a main body of 50 wt% graphene to construct the conductive network. Subsequently, the film was subjected to a simple and fast Joule heating treatment. The polyimide was treated to generate a similar sp^2^-hybridised covalently bonded carbon atom structure with graphene as a template, which enhanced the conductive properties of the graphene/polyimide films by ~76.85%.

## 2. Methods

Graphene was bought from Shanghai Kington Co., Ltd., Shanghai, China. (SDP 600, with a flake size of 200–800 nm, a layer thickness of 3–10 nm, and an O/C ratio < 5:95) [35]. 4,4′-diaminodiphenyl ether (ODA), 1,2,4,5-Benzenetetracarboxylic anhydride (PMDA) and Dimethylacetamide (DMAc) were brought from China National Pharmaceutical Group Co., Ltd., Shanghai, China.

### 2.1. Synthesis

**Figure 1:**
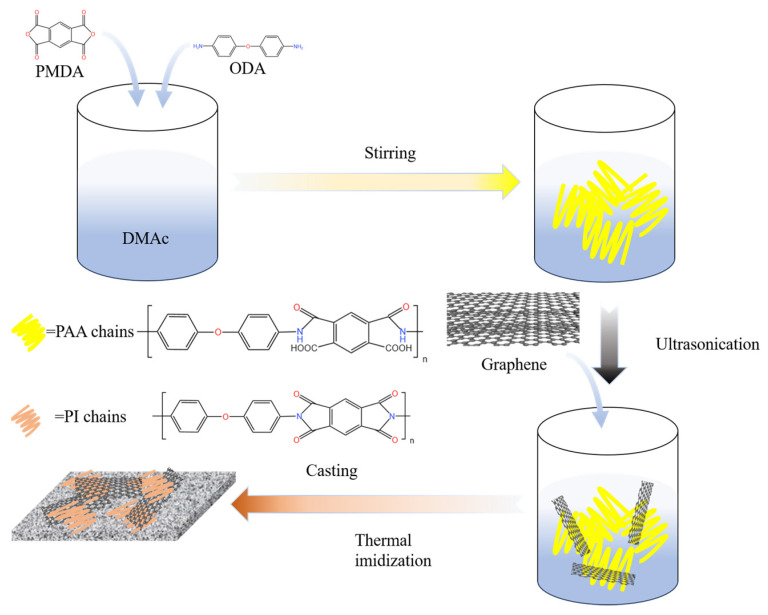
Schematic diagram of the preparation process of graphene/polyimide film.

A total of 2.0 g of ODA (0.01 mol) and 2.18 g of PMDA (0.01 mol) were added to 31 mL of DMAC, and the mixed solution was stirred vigorously under a nitrogen stream for 24 h at room temperature to obtain a solution of the polyimide precursor, polyamic acid (PAA) [36]. A total of 4.18 g of graphene was then added to this solution and stirred for 1 h at room temperature to obtain a graphene/PAA mixed suspension. The graphene/PAA solution was poured onto a 200 mm × 200 mm steel plate substrate, which was then degassed in a vacuum for 10 min, placed in a circulating air oven and heated to 350 °C at a rate of 1 °C/min for thermal imidisation and, then, held for 12 h and cooled to room temperature within the oven [36] (Figure 1).

### 2.2. Joule Heating Treatment

The resulting graphene/polyimide film was cut into long strips of 40 × 10 × 0.1 mm, connected to a DC power supply at both ends under a protective nitrogen atmosphere, with the current being increased at a rate of 0.1 A/2 min in a constant current mode until the power was reduced [32].

**Figure 2:**
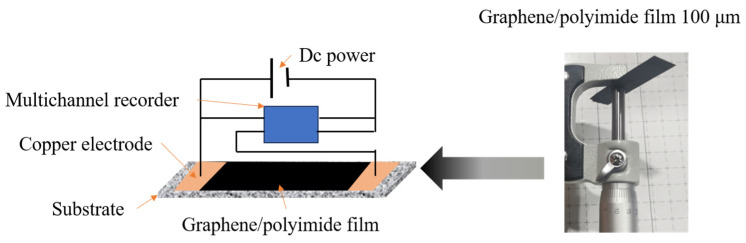
Graphene/polyimide film sample and schematic diagram of the Joule heating treatment of graphene/polyimide film (The substrate is a high-temperature-resistant aluminium oxide ceramic. Graphene/polyimide film is placed on the substrate, covered with copper electrodes and clamped with fixtures).

The thickness of the resulting graphene/polyimide film was approximately 100 μm (Figure 2). There were two samples: the raw sample without the Joule heating treatment (Raw) and the sample with a complete Joule heating treatment (7.0, named after the maximum supply of output current achieved by the Joule heating treatment, which was 7.0 A). In addition, the samples that had undergone the Joule heating treatment but not completely were selected and named 2.5, 4.0 and 5.5 (after the maximum supply output currents achieved, which are 2.5 A, 4.0 A and 5.5 A, respectively).

All the measurements were performed on the graphene/polyimide samples, sampling 3–5 times for each test. The sheet resistance was measured by a four-probe sheet resistor (Helpass 2523, Helpass, manufactured by Helpass Electronic Technology Co., LTD, Changzhou, China). The surface morphology was observed using a scanning electron microscope (Sigma 300, Zeiss, manufactured by Carl Zeiss AG, Oberkochen, Germany, acceleration voltage 10 kV). The morphology and structure were analysed with a transmission electron microscope (JEM-2100F, JEOL, manufactured by JEOL (BEIJING) Co., Ltd., Beijing, China, acceleration voltage 100 kV). The structure and atomic valence states were carefully analysed by X-ray photoelectron spectroscopy (K-Alpha, Thermo, manufactured by Thermo Fisher Scientific (China) Inc., Shanghai, China). The band structure was characterised by ultraviolet photoelectron spectroscopy (Escalab 250Xi, Thermo, manufactured by Thermo Fisher Scientific (China) Inc., Shanghai, China, 3 eV). The structure was analysed by X-ray diffraction using an X-ray diffractometer (XRD-6100, Shimadzu, manufactured by Shimadzu (China) Co., LTD, Shanghai, China, copper target, speed 2.0000 (deg/min)). The Raman spectra were also analysed using a confocal Raman fast imaging microscope (Alpha 300R, WITec, manufactured by Oxford Instruments Group, Ulm, Germany, wavelength 488 mm). The crystallinity and diffraction pattern were analysed by wide-angle X-ray scattering (Xeuss 3.0, Xenocs, manufactured by Xenocs (Suzhou) Scientific Instrument Co., LTD, Suzhou, China, distance 50 mm, wavelength: 1.54189 Å). The electrochemical impedance spectroscopy was analysed by an electrochemical workstation (CHI-760E, Chinstruments, manufactured by Shanghai Chinstruments Instrument Co., LTD, Shanghai, China).

## 3. Results and Discussion

The sheet resistance of the raw graphene/polyimide film samples without the Joule heating treatment and the samples of sizes 2.5, 4.0, 5.5 and 7.0 was measured using four-probe resistivity measurements. Each sample was tested three times, and the results are shown below (Table 1).

The conductivity of the sample can be calculated by the formula [37,38]:

(1) $$\sigma =\frac{1}{\rho }=\frac{1\;}{R\times T}=\frac{1\;}{R\times s÷L}$$

where σ is the conductivity; ρ is the resistivity; R is the measured resistance; T is the thickness of the sample; s is the cross-sectional area of the sample; and L is the length of the sample.

The electrical conductivity of the pristine graphene/polyimide film was calculated to be ~1484.79 s·m^−1^, while the treated graphene/polyimide film had a conductivity of ~2625.84 s·m^−1^, an enhancement of ~76.85%.

**Figure 3:**
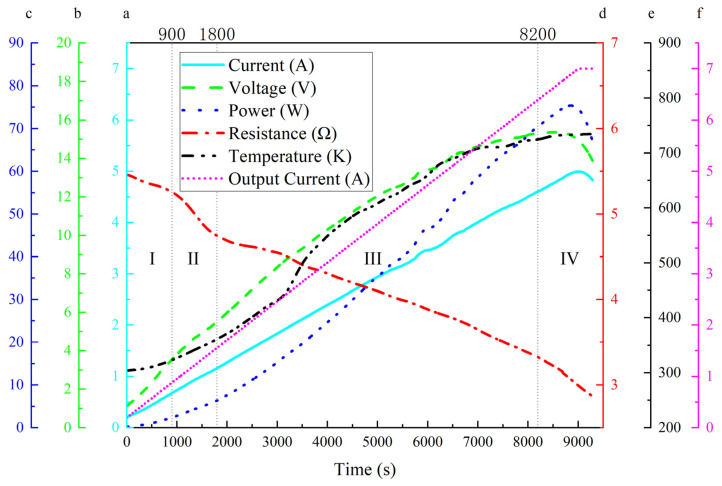
Plot of Joule heating treatment process versus time, with different test objects: (a) current, detected through the sample; (b) voltage, detected through the sample; (c) power, detected through the sample; (d) resistance, detected through the sample; (e) temperature, detected through the sample; and (f) output current, the output current of DC power supply; the Roman numerals indicated the different periods during the Joule heating treatment.

During the Joule heating treatment of the graphene/polyimide films, the input electrical energy was partly converted into thermal energy by the electrothermal effect and partly served as the driving energy for the rearrangement of the atoms in the material. As a result, when the atomic rearrangement was near completion, the power of the material showed a significant decrease as the current continued to increase, as shown in Figure 3. The current through the sample was less than the output current of the power supply due to the circuit design and the multichannel recorder; this difference increased as the current increased. It is worth noting that while the output current remained at 7.0 A, the current through the sample decreased. This phenomenon may have resulted from the decrease in the shunt of the current through the sample due to the decrease in the resistance. From Figure 3, it can be observed that the Joule heating process can be divided into four periods: period I, from the start to ~900 s, showing a slow decrease in resistance and relatively fast increases in voltage; period II, from ~900 s to ~1800 s, with an obvious, fast decrease in resistance; period III, from ~1800 s to ~8200 s, with a slow decrease in resistance, a gradually declining rate of voltage increase, and a variational increase in temperature, which inferred a complex collision process between the electrons and the polyimide chains; period IV, from ~8000 s to the end, during which resistance showed an accelerated decrease, voltage, power and current sequentially reached their peaks and then declined, and the increasing temperature trend flattened. This pattern suggests that the change in the internal resistance of the material is produced by multiple factors. An important possible influencing factor is the combined effect of current and heat, causing the excitation of more charge carriers in the graphene/polyimide film as the temperature increases, leading to the phenomenon of a decreasing resistance with increasing temperature. In this study, in the first and third periods, the resistance decline in the graphene/polyimide film was mainly attributed to the temperature increase, which was due to the semiconductor properties of the graphene/polyimide film material, a combination of the quite-high and zero bandgaps of polyimide and graphene, respectively. In the second period, the main influence may have been the atomic rearrangement phenomenon due to the movement of electrons driven by the electric current. The recrystallisation of carbon atoms in the presence of electrons greatly improved electron transport within the material, resulting in a rapid decrease in resistance. The fourth period may have been dominated by the reduction in resistance caused by the large-scale formation of a new graphene/polyimide interface near the end of the atomic rearrangement process. The conductivity measured by the Joule heating method was significantly different from that measured by the four-probe method, mainly due to the resistance change caused by the inevitable temperature increase during Joule thermal experiments. The rest might have been caused by the different contact resistance of the two measurement methods.

During the Joule heating treatment, according to Equation (1), the conductivity of the raw sample was ~7328.69 s·m^−1^, and that of the sample after Joule heating was ~13,907.28 s·m^−1^, which was an increase of ~89.76%. This difference, compared to the conductivity registered during the sheet resistivity calculations, may have been due to the current and temperature effects.

**Figure 4:**
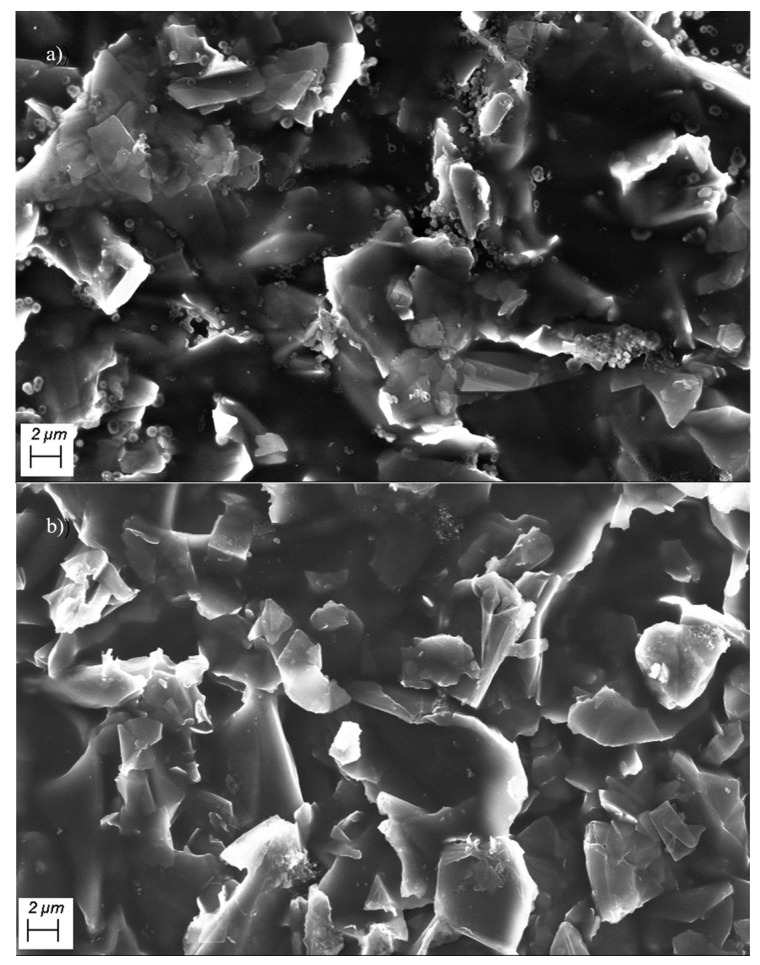
SEM of (**a**) raw graphene/polyimide films and (**b**) graphene/polyimide films after Joule heating treatment.

Figure 4a,b show scanning electron microscope (SEM) images of the graphene/polyimide films before and after treatment. As can be seen from the figure, the graphene sheets are stacked together, and, on them, there are irregular gelatinous polyimides. From the structure of graphene/polyimide films at the same scale, the Joule heating treatment did not damage their integrity, as there were no obvious holes and shrinkage. Meanwhile, the more detailed microstructure was observed by transmission electron microscopy.

**Figure 5:**
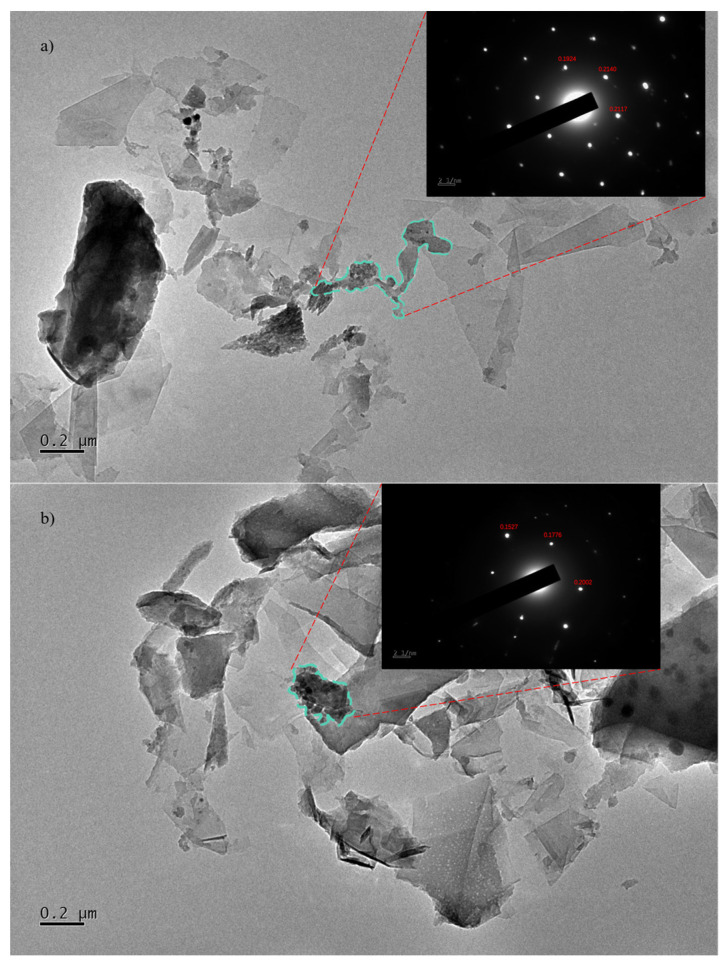
TEM and SAED of (**a**) raw graphene/polyimide films and (**b**) graphene/polyimide films after Joule heating treatment (the calibrated numbers indicated the crystal plane’s spacing).

Figure 5a,b include the transmission electron microscope (TEM) images of the graphene/polyimide films before and after treatment. The samples were dispersed with ethanol, treated ultrasonically and, then, placed onto the copper grid. As can be seen in Figure 5a, the graphene was stacked in a lamellar state, and it maintained a relatively complete and clean edge. Between its lamellae, the graphene was bonded by polyimide. For the selected region electron diffraction (SAED) pattern of the connected parts, a new crystalline diffraction pattern like graphene but with different lattice parameters appeared. This diffraction pattern indicated that the graphene/polyimide thin-film material formed, through the crystallisation of polyimide and using graphene as a template, a new phase resembling a six-membered ring structure during the Joule heating treatment [39]. By calibrating the crystal plane’s spacing, it can be seen that the last three spacings of this new phase (0.1527 nm, 0.1776 nm and 0.2002 nm) were smaller than those of the raw sample’s graphene (0.1924 nm, 0.2140 nm and 0.2117 nm).

**Figure 6:**
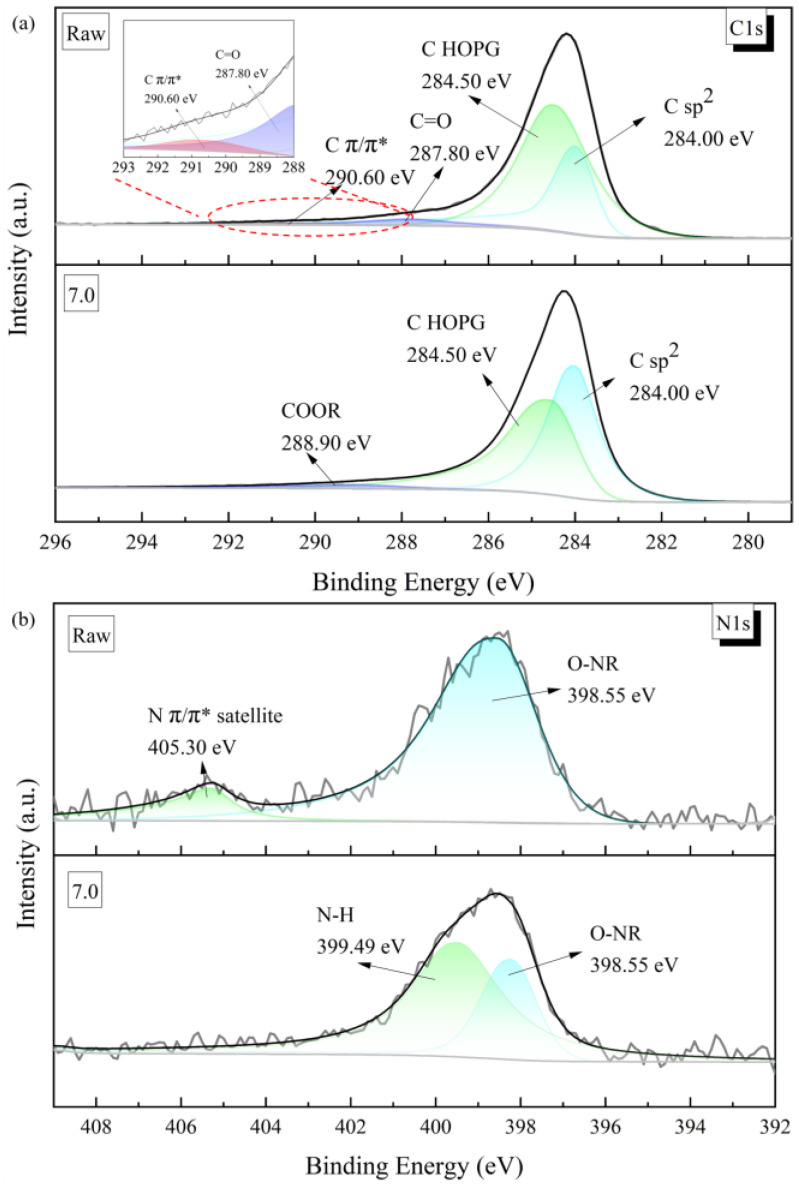
(**a**) XPS of graphene/polyimide films before and after Joule heating treatment, C1s peak; (**b**) XPS of graphene/polyimide films before and after Joule heating treatment, N1s peak (different shades represented different chemical bonds).

**Figure 7:**
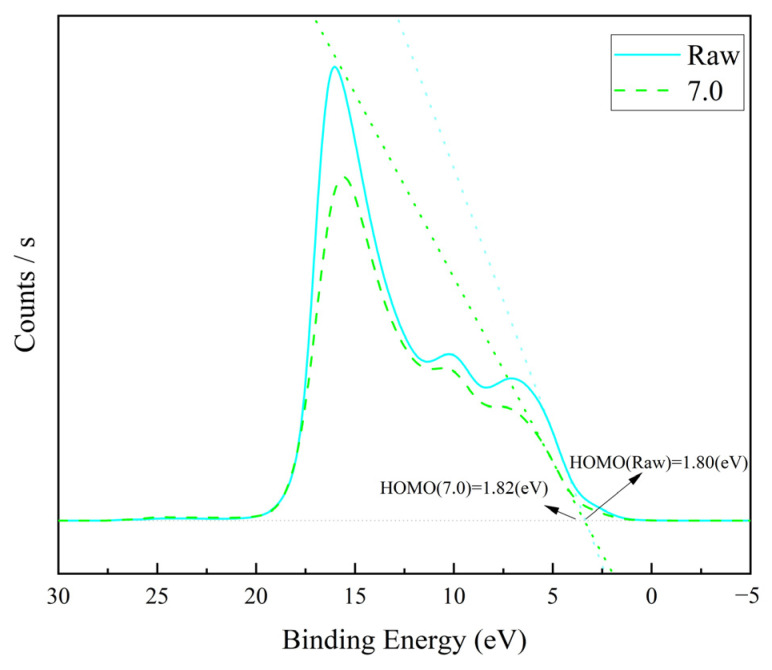
UPS of graphene/polyimide films before (Raw) and after Joule heating treatment (7.0).

This structural change in the thin-film material was also evident in its electronic structure. As can be seen from the C1s peaks of the materials characterised by X-ray photoelectron spectroscopy (XPS), the graphene/polyimide films both before and after treatment showed clear characteristics of inorganic carbon materials, with the peaks being sharp and asymmetric overall (Figure 6a). Both of them were characterised by the C sp^2^ characteristic peak (284.00 eV), with a highly oriented graphite-like crystal structure. After Joule heating, the C Π/Π* bond (290.60 eV), which was present in the sample in small amounts, disappeared, and a COOR bond (288.90 eV) appeared, which may have been related to the oxidisation of the C=O bond (287.80 eV) in the original material [40]. Correspondingly, the N1s energy spectra of both materials showed a clear signal from the imide ring (O-NR, 298.55 eV) (Figure 6b). However, in the original material, there was also a more pronounced peak at 405.30 eV caused by the signal drift in the Π-bond of N in the imide ring. The X-ray photoelectron spectroscopy results indicated some loss and rearrangement of the chemical bonds of C. The recombination of these bonds could have come from the electrons moving, under the influence of an external power source, in the direction of the potential difference, accompanied by the perturbation of the Π-electronic structure inside the material, during the Joule heating treatment. In this process, the electrons would have been driven by the voltage and left the domains throughout the material space, with the consequent re-bonding of the atoms, to form a more stable and oriented crystal structure. From the perspective of the energy bands, as shown in Figure 7, the HOMO orbits of the graphene/polyimide films before and after the Joule heating treatment were close, both about 1.81 eV. Moreover, the band structure of the two was similar, revealing that the Joule heat treatment did not change the band structure of the graphene/polyimide films.

**Figure 8:**
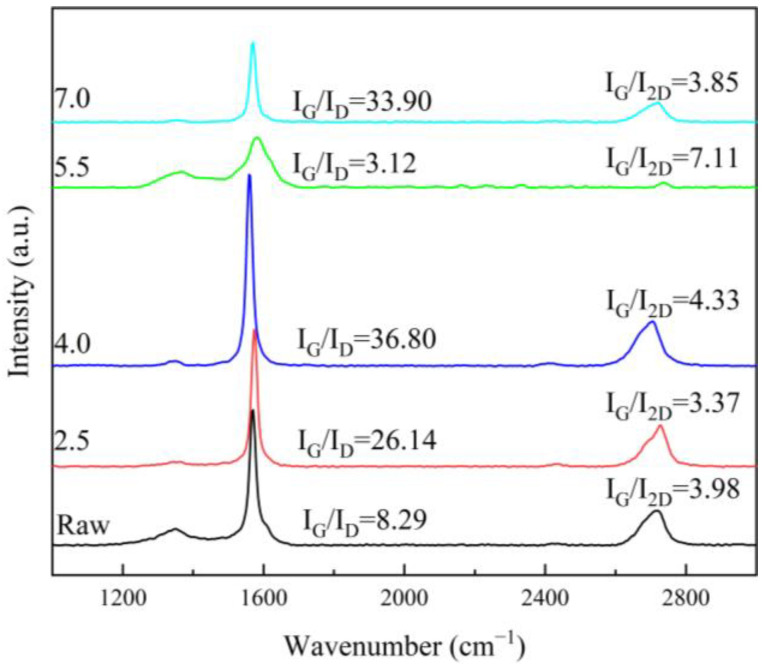
Raman spectra of graphene/polyimide films before, in and after Joule heating treatment.

**Figure 9:**
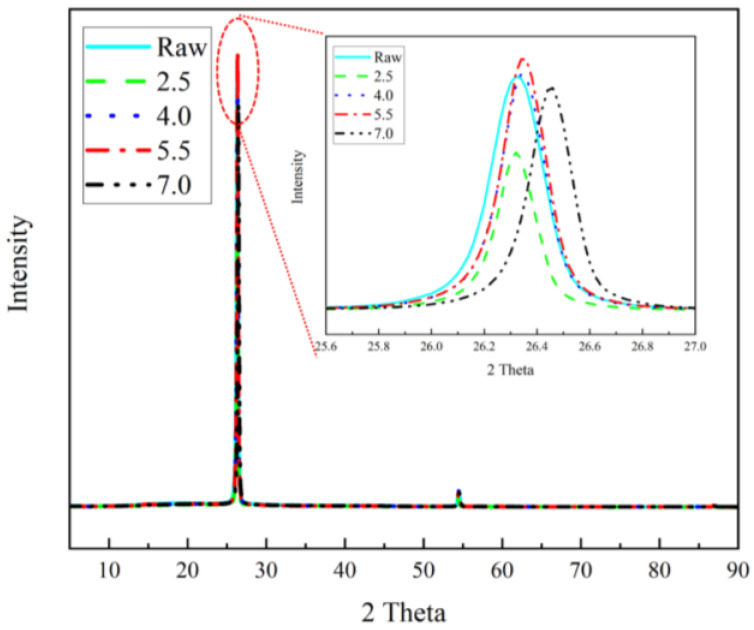
XRD pattern of graphene/polyimide films.

The Raman spectroscopy results of the films showed that the raw samples, the samples during the treatment process, and the already-treated samples exhibited graphene-like spectral features (Figure 8) [41]. In particular, three major peaks—D, G and 2D—were found in all the samples, located at ~1350 cm^−1^, ~1570 cm^−1^ and ~2700 cm^−1^, respectively. The D peaks are usually considered to be defect or edge peaks of graphene, involving a process of inelastic intervalley scattering of an iTO phonon near the K point with the intervalley scattering of a defect, which would activate the respiratory vibrational modes of the six-membered ring. The G peak is usually considered to be a characteristic peak of sp^2^-hybridised carbon atoms, and it involves the intravalley scattering process of two doubly condensed iTO and iLO phonons near the Γ point. The peak generally arises from an in-plane vibration of the sp^2^ carbon atom, corresponding to a vibrational mode with E_2g_ symmetry. The 2D peak, on the other hand, is produced by two-interval inelastic scattering of troughs from an iTO phonon near the K point, and it is an overtone of the D peak. The 2D band is the second-order D band corresponding to the two phonon lattice vibrations, and it is a characteristic peak of graphene’s structure [42]. The number of graphene layers can be inferred from the ratio of G/2D band intensities (I_G_/I_2D_), as well as the shape and position of these bands [41]. As shown in Figure 8, the 2D band was not sharp, and the I_G_/I_2D_ value was relatively big, indicating that the graphene sample had a low degree of graphitisation and significant layer-stacking. From the change in the value of I_G_/I_2D_, the stacking degree of graphene became larger at 4.0 and 5.5 A. This may have been due to the appearance of a new crystalline phase caused by atomic rearrangement during the Joule heating process, which reduced the inelastic cavity scattering of iLO phonons. The new phase took graphene as a template, showing a greater degree of stacking. Then, with the growth of the new crystalline phase, the ratio of I_G_/I_2D_ tended to return to the raw sample’s value. The D’ peak at ~1620 cm^−1^ was present in the raw sample. The formation of this peak mainly involved a process of inelastic intravalley scattering of an iLO phonon near the K point, with the intravalley scattering of a defect, suggesting a defect in the raw sample, that disappeared with the Joule heating treatment. It is noteworthy that the G peak first appeared to be red-shifted during the treatment, implying that, in the graphene/polyimide films, during the Joule heating process, the rearrangement of the atomic structure caused by the electrons’ directional movement led to a recrystallisation phenomenon. The increase in crystallinity led to an expansion of the material, resulting in a decrease in the frequency of the phonons interacting with the incident photons. The subsequent blue shift, on the other hand, indicated the formation of a large number of new interfacial layers between the graphene and the recrystallised polyimide, which had a higher phonon frequency. The G peak of the final 7.0 sample showed another small red shift, indicating the gradual stabilisation of the new interface and a slight increase in crystallinity. This phenomenon was also confirmed by the X-ray diffraction (XRD) results (Figure 9), in which it was clear that the main peak of the film was located at the position of the (002) peak and that 2θ was close to 26.4°. It could be seen that, with the Joule heating treatment, the 2θ angle of its Schott peak became larger, indicating smaller lattice spacing and the expansion of the material. Moreover, the treated sample had the largest 2θ angle, which was subject to the double effect of recrystallisation and the formation of a new interfacial layer.

The average grain size ($L_{a}$) in the graphene/polyimide films could be calculated from the intensity ratio of the G and D peaks in the Raman spectrum (Figure 8) [43], as follows:

(2) $$L_{a}=\left(2.4\times 10^{-10}\right)\times \lambda^{4}\times \frac{I_{G}}{I_{D}}$$

where λ is the wavelength of the incident light in the Raman spectrum. From this result, the average grain size in the graphene/polyimide films appeared to increase, then decrease and increase again during the Joule heating treatment, which was supported by the shift in the Raman peak position. Furthermore, the growth of new grains followed the Arrhenius equation [43], as follows:

(3) $$k=A\exp\left(-\frac{E_{A}}{RT}\right)$$

where k is the reaction rate of grain growth at a given temperature; A is the prefactor, also known as the Arrhenius constant, in the same units as k; $E_{A}$ is the experimental activation energy in J·mol^−1^; R is the molar gas constant, =8.314 J·K^−1^mol^−1^; and T is the reaction temperature.

From the voltage–temperature curve in Figure 1, it can be seen that the range and variation in the reaction temperature was large, so the correction of Equation (3) was obtained [44]:

(4) $$k=A{\left(\frac{T}{T_{0}}\right)}^{n}\exp\left(-\frac{E_{A}}{RT}\right)$$

where n is the experimentally measured constant; T is the reaction temperature; and $T_{0}$ is the initial reaction temperature. Molecular dynamics calculations show that the temperature at which PI crystals form graphene rings is ~2400 K, and the critical temperature for PI carbonation is around 700 °C [26]. Therefore, the present graphene/polyimide film did not form graphene rings nor undergo large-scale carbonation, and the whole should have still retained the structure of polyimide, with only some minor atomic rearrangements to form new grains and crystal phases. In this process, graphene improved the aggregation structure of polyimide molecules, forming a crystal structure which was more conducive to electron transport.

**Figure 10:**
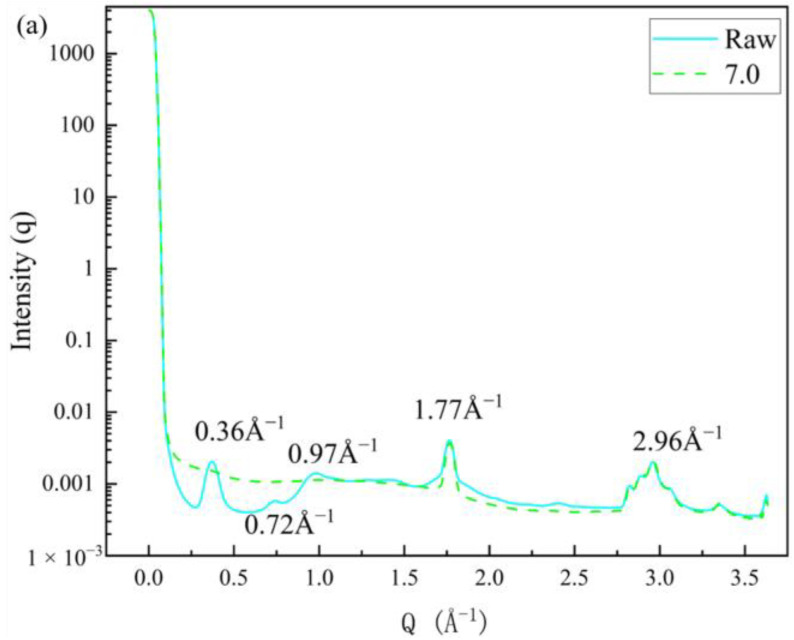

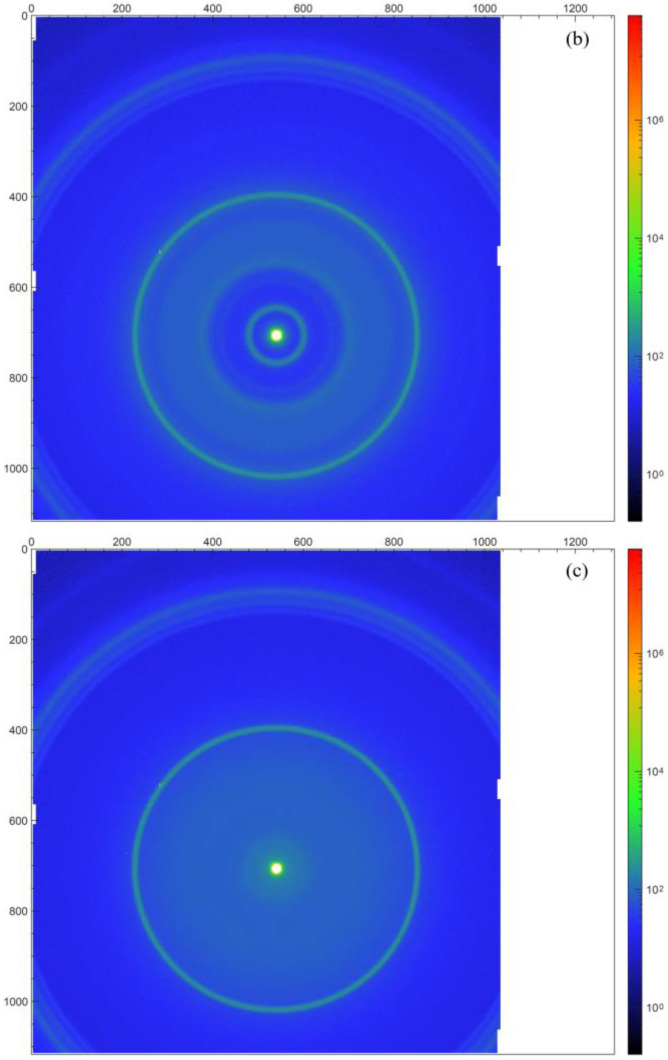
Wide-angle X-ray scattering of the graphene/polyimide film before and after Joule heating treatment: (**a**) 1D pattern; (**b**) 2D diffraction picture of the untreated pristine graphene/polyimide film; (**c**) 2D diffraction picture of the graphene/polyimide film after Joule heating treatment.

The detailed microstructure formed by this localised atomic rearrangement was difficult to capture directly, but it was possible to characterise some of the microstructural differences in the graphene/polyimide thin films before and after Joule heating by wide-angle X-ray scattering (WAXS). The 1D WAXS pattern in Figure 10a shows the diffraction rings at 1.77 Å^−1^ and 2.96 Å^−1^ for the graphene/polyimide films both before and after Joule heating. They correspond to d-space values of 3.55 Å and 2.12 Å, respectively, with the former being due to the spacing of the few graphene stacks in the sample and the latter being close to the lattice constant of graphene. The untreated pristine graphene/polyimide film also had three diffraction loops of 0.36 Å^−1^, 0.72 Å^−1^ and 0.97 Å^−1^, corresponding to d-space values of 17.45 Å, 8.73 Å and 6.48 Å, respectively. The two sites at 17.45 Å and 8.73 Å were derived from the same lamellar-stacking spacing structure between the main chains of the polyimide polymer chain caused by the separation of the side-chains. Moreover, the site at 6.48 Å was likely to have been derived from the imide ring. This result suggested that, during the Joule heating treatment, the side-chain structures of some polyimide polymer chains and the imide ring were destroyed and their atomic structures rearranged, resulting in the disappearance of the diffraction rings at these locations. In addition, the diffraction rings of the pristine graphene/polyimide films at 1.77 Å^−1^ had a half-peak width slightly larger than that of the graphene/polyimide films after the Joule heating treatment. This indicated that the crystallinity of the graphene in the samples increased after Joule heating.

**Figure 11:**
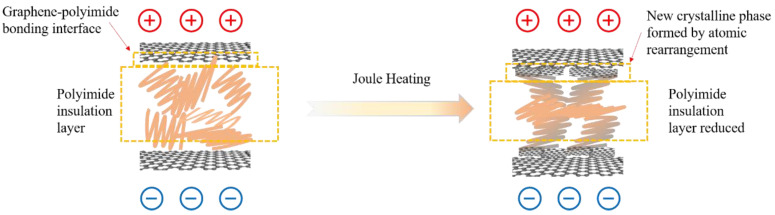
Schematic representation of electron transport and structural changes in graphene/polyimide films under Joule heating treatment.

From the above results, it can be inferred that, in the graphene/polyimide film, the graphene lamellae are connected by polyimide, forming a microstructure similar to a double-layer capacitor with graphene as the electrode plate and polyimide as the insulator. As shown in Figure 11, when a current is applied, the graphene is polarised at both ends. At this point, when the graphene is polarised to a certain extent, a pathway for electron transport is formed, due to the tiny size of the polyimide, coupled with a small number of out-of-domain electrons from the benzene ring on it. When the graphene/polyimide film is subjected to a Joule heating treatment, the crystallinity of the graphene increases. The polyimide does not undergo significant pyrolysis as there are no obvious shrinkage and holes in the films after Joule heating, as shown in Figure 4, coupled with the fact that no noticeable gas release can be observed during the treatment. The structural changes in the graphene/polyimide films during a Joule heating treatment may be mainly due to the breaking of ether bonds, benzene rings and imide rings [45] which, once broken, are oriented, to some extent, along the direction of the current. Finally, under the influence of the current, a certain number of rearrangements of the atoms exposed to the current occurs, using the graphene at the interface as a template. On the one hand, this reduces the distance between the graphene in the capacitive structure of the bilayer, thus reducing the resistance. On the other hand, the rearranged atoms that use graphene as a template will provide more off-domain electrons, thus increasing the number of charge carriers. With this synergistic effect, the electron mobility of the graphene/polyimide film is greatly increased after treatment with Joule heating.

**Figure 12:**
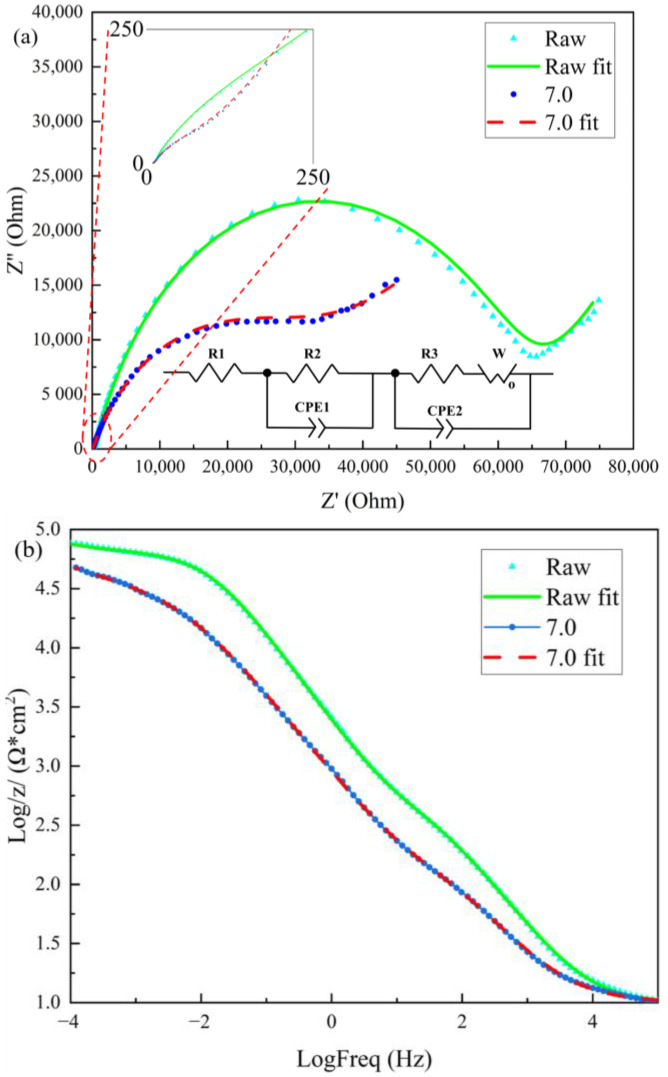

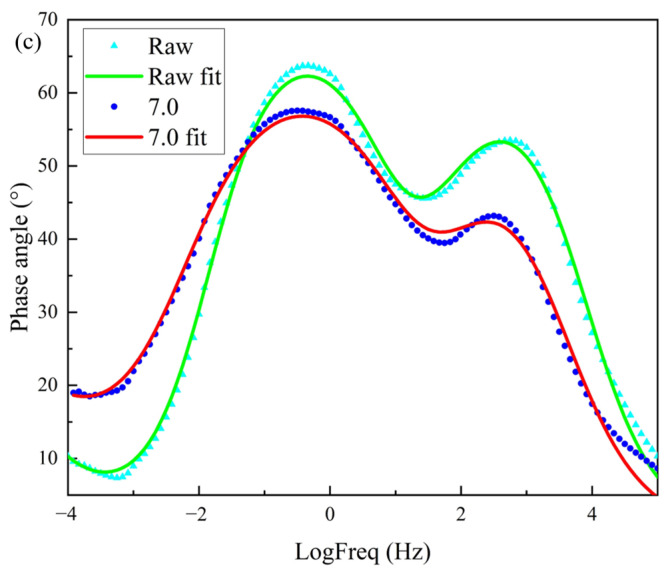
Electrochemical impedance spectroscopy of graphene/polyimide films: (**a**) Nyquist spectroscopy and equivalent circuit diagram; (**b**) Bode value spectroscopy; (**c**) Bode phase angle spectroscopy.

The above conclusion can be illustrated using electrochemical impedance spectroscopy (EIS). As shown in Figure 12, a complex Faraday impedance appears when an AC signal passes through the graphene/polyimide film. In the low-frequency part, the diffusion effect exceeds the electrochemical effect, and a Warburg impedance appears. Meanwhile, in the high-frequency part, part of it manifests as a capacitive arc of the bilayer capacitance, and the other part is the relaxation of the surface state in response to the potential due to the complexity of the graphene/polyimide film interface. The Faraday current appears as a second time constant due to this surface state factor. Moreover, this time constant exhibits capacitive impedance. The electrochemical impedance characteristics of graphene/polyimide films before and after a Joule heating treatment are more consistent. As can be seen in Figure 12a, both of the semicircles of the raw film were larger than those of the film treated with Joule heating in the Nyquist plot, probably due to the electron transfer impedance and the capacitive impedance of the interfacial time relaxation of the raw film both being larger than those of the treated film. Moreover, because of the larger capacitive reactance, the slope of the straight line representing the Warburg impedance of the raw film in the Nyquist plot was greater. In the Bode plots’ modulus value plots in Figure 12b, the modulus values of the raw films were always larger than those of the treated films. Moreover, in the low-frequency region, at frequencies <10^−2^, the modulus value of the raw film remained almost constant, while the modulus value of the treated film increased with decreasing frequencies, probably due to the greater diffusion of the treated film. In the Bode plot’s phase-angle plot in Figure 12c, two peaks appeared for both the raw and treated films, indicating the presence of two time constants for both. The first time constant in the high-frequency region >10^2^ may have resulted from the impedance of electron transfer within the sample. The second time constant in the mid-frequency range <10^0^ may have arisen from the capacitive impedance of the graphene–polyimide interface.

Their equivalent circuits are shown in Figure 12a, and the original parameters of each equivalent circuit are shown in Table 2. It can be seen that the internal resistances of the samples before and after treatment were quite close to one another (R1: 9.847 and 10.00, respectively). Their constant phase-angle components, CPE-P, on the other hand, were all around 0.7 (CPE1-P: 0.71187 and 0.66545, CPE2-P: 0.77232 and 0.69409, respectively), indicating that diffusion effects were present in the samples and that Warburg impedance appeared. While CPE-T represents the electrochemically active area of a capacitor, both CPE-T model parameters of the treated samples were almost three times those of the original samples (CPE1-T: 0.00006259 and 0.00019798, CPE2-T: 0.00010222 and 0.00032375, respectively). With respect to the electron transfer impedance in the sample’s Faraday impedance, the R2 (309.10 and 88.32, respectively) and R3 (64,175 and 23,741, respectively) of both of the treated samples’ capacitive impedances were only 1/3 of those of the original samples, indicating that the distance of the electrons travelling through the polyimide was much shorter. In the Warburg impedance, the W-P of the two samples was closer (W-P: 0.26776 and 0.20536, respectively), indicating the existence of Warburg impedance in both samples. W-R (W-R: 383.6 and 30,882.0, respectively) is the coefficient of Warburg impedance in an equivalent circuit. W-T is the diffusion flux of a sample, which is given by the formula L^2^/d, where L is the thickness of the diffusion layer, and d is the diffusion coefficient [46]. The W-T of the treated sample was more than 2500 times that of the original sample (W-T: 1.507 and 3849.000, respectively). This indicated that the thickness of the diffusion layer containing the electrons involved in the reaction was significantly greater than that of the original sample’s layer when the electrons overcame the electron transfer impedance to reach the surface of the sample and diffused by reacting with the ions in the electrolyte in samples treated with Joule heating. In the electrochemical equivalent circuit, R1 was the systematic internal resistance of the electrochemical test system. The first Faraday impedance may have been the capacitive impedance in the graphene/polyimide two-electrode capacitance model. The decrease in R2 may have been due to the reduction in the thickness of the polyimide layer in the model. The second Faraday impedance may have originated from the graphene–polyimide junction interface. The decrease in R3 suggested a decrease in the interface impedance. Moreover, the large increase in W-T suggested an increase in the thickness of the interface capable of electron transfer. Meanwhile, the increase in the electrochemically active area represented by CPE-T predicted an active interfacial reaction. These conclusions are consistent with the above-described reaction model inference.

**Figure 13:**
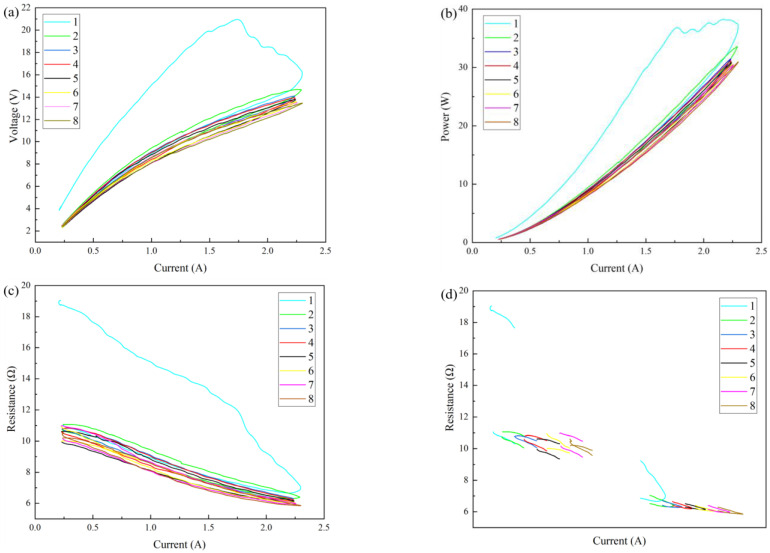
Joule heating treatment on graphene/polyimide films with eight cycles: (**a**) current—voltage curve; (**b**) current—power curve; (**c**) current—resistance curve; and (**d**) current—resistance terminal point curve.

The effect of a Joule heat treatment on the structure of graphene/polyimide films is irreversible. Moreover, multiple Joule heat treatments can further improve the conductivity of graphene/polyimide films, but the overall effect shows a slowing trend. Figure 13a shows the CV curves of the graphene/polyimide films treated with eight Joule thermal cycles. As can be seen from the figure, in the first cycle’s current increase stage, the voltage dropped significantly with the increase in the current. However, according to the CP curve (Figure 13b), the voltage drop was not synchronised with the power drop. This verified that, under the action of Joule heating, the voltage provided by the external power supply helped the atoms in the graphene/polyimide film to break away from their original positions and rearrange across the energy barrier, which was macroscopic, as the maximum voltage. After that, the electrical energy continued to drive the atoms to complete the rearrangement, so the power continued to increase, while the voltage decreased. This process was not instantaneous and did not occur synchronously throughout the material, so it appeared as a fluctuation in voltage and a wave-like rise in power. According to the current–resistance curve (Figure 13c), during the first cycle’s current increase stage, the resistance showed an obvious four-stage decline. However, in the first, second and eighth cycles’ current reduction stages, the resistance showed a slow negative correlation with the current change. Specifically, it can be seen from the current–resistance endpoint curve (Figure 13d) that the resistance was not stable at the beginning and at the end of cycles 2–8. However, when the current of the second to eighth cycles was at a maximum, the resistance was relatively stable, showing a phenomenon of successive reduction, and the reduction trend slowed down. This may have been because, under the synergistic effect of electric heating, a carrier is more excited with an increase in the number of cycles when the current is at a maximum. At the beginning and at the end of a cycle, electron–hole coordination may occur due to the reduced driving force of the excited carrier. However, this process is relatively random, so the resistance at the beginning and at the end of a cycle is unstable. In general, Joule heating changes the atomic structure at the interface of the graphene/polyimide films, causing it to rearrange and form a crystal structure which is more conducive to electron transport. This process is most obvious during the first Joule heat treatment. Moreover, with the increase in Joule heat treatments, the improvement in the films’ conductivity tends to slow down. After the eighth cycle, the conductivity is ~2879.97 s·m^−1^, an increase of ~93.94%. It can be inferred that, after a certain number of Joule heating treatments, the interface structure of graphene/polyimide films will be fixed and no longer change with the input current, at which point the conductivity of the graphene/polyimide film would reach its maximum. The sheet resistivity of graphene/polyimide film with eight Joule heating treatment cycles is shown in Table 3.

## 4. Conclusions

This work demonstrated a new approach to improve electron transfer between graphene lamellae, with the development of graphene’s electrical properties. The results showed that graphene/polyimide composite films exhibited a significant increase in electrical conductivity of around ~76.85% under a Joule heating treatment. A series of characterisation results indicated that this change in performance may have been due to the rearrangement and recrystallisation of polyimide atoms at the graphene/polyimide interface. During the Joule heating treatment, the polyimide molecules, with a graphene template, underwent atomic rearrangement under the action of an electric current, forming more regular phases favourable to electron transport. Eight experimental cycles showed that this structural change was irreversible and could be further completed by multiple Joule heating treatments, reaching a final conductivity improvement of ~93.94%. Accordingly, the researchers proposed a model for the microstructure of a double-layer capacitor with a graphene electrode plate and a polyimide insulating medium. The model revealed that the Joule heat treatment reduced the distance between the graphene sheets and increased the number of off-domain electrons, thus enlarging the diffusion region for electron reactions. This work provided a feasible approach to better utilise graphene’s excellent electrical conductivity in practice, key to the application of many graphene composites. This study can help researchers in their efforts to achieve better graphene performance in areas such as electrical and thermal conductive materials. In addition, it provides a viable idea for polyimide-induced carbonation.

## Figures and Tables

**Table 1:** The sheet resistance of graphene/polyimide film samples.

Samples	The Sheet Resistivity (Ohms/Square)			
	No. 1	No. 2	No. 3	Mean
Raw	6.67152	6.66236	6.87105	6.73498
2.5	6.35767	6.38833	6.29538	6.34127
4.0	6.09058	6.26109	6.29049	6.21405
5.5	5.61149	5.85083	5.78398	5.74877
7.0	3.93159	3.68096	3.81237	3.80831

**Table 2:** Equivalent circuit element parameter of graphene/polyimide film electrochemical impedance.

	Raw Graphene/Polyimide Film	Joule Heating Treated Graphene/ Polyimide Film
R1	9.847	10.000
R2	309.10	88.32
CPE1-T	0.00006259	0.00019798
CPE1-P	0.71187	0.66545
R3	64,175	23,741
W-R	383.6	30,882.0
W-T	1.507	3849.000
W-P	0.26776	0.20536
CPE2-T	0.00010222	0.00032375
CPE2-P	0.77232	0.69409

**Table 3:** The sheet resistance and conductivity of graphene/polyimide film with eight Joule heating treatment cycles.

Cycles	The Sheet Resistivity (Ohms/Square)	Conductivity (s·m^−1^)
Raw	6.73498	1484.97
1	3.80831	2625.84
2	3.69404	2707.06
3	3.62392	2759.44
4	3.57388	2798.08
5	3.53197	2831.28
6	3.51127	2847.97
7	3.49358	2862.39
8	3.47226	2879.97

## Data Availability

Due to privacy restrictions, the study data cannot be made public. Please contact the corresponding author if you have any questions.
